# Unlocking Gut Health: The Potent Role of Stilbenoids in Intestinal Homeostasis

**DOI:** 10.3390/ani15030417

**Published:** 2025-02-03

**Authors:** Tiantian Meng, Ziwei Wen, Xiaofang Cheng, Cencen Li, Pengpeng Zhang, Dingfu Xiao, Yongjie Xu

**Affiliations:** 1 College of Life Science, Xinyang Normal University, Xinyang 464000, China; meng-tiantian@foxmail.com (T.M.); wzw1452861647@163.com (Z.W.); xfchengsunny@163.com (X.C.); licencen2009@126.com (C.L.); ppzhang15@163.com (P.Z.); 2 Yuelushan Laboratory, College of Animal Science and Technology, Hunan Agricultural University, Changsha 410128, China

**Keywords:** stilbenoids, biological activities, gut health, immune responses, gut microbiota

## Abstract

Stilbenoids are a class of naturally occurring phenolic compounds found in various plant species, characterized by a stilbene backbone with diverse substituents that confer a range of biological activities. These compounds exhibit antioxidant, anti-inflammatory, and antimicrobial properties, making them promising candidates for improving intestinal health. The intestinal tract plays a critical role in nutrient digestion, absorption, and immune defense, and maintaining its integrity is vital for animal growth. Stilbenoids contribute to gut health by enhancing intestinal morphology, supporting mucosal immune responses, regulating gut microbiota composition, modulating metabolic pathways, and maintaining mitochondrial health. This review provides a comprehensive analysis of key stilbenoids, including resveratrol, pterostilbene, piceatannol, and oxyresveratrol, focusing on their biological effects and regulatory mechanisms. By highlighting their roles in mitigating intestinal inflammation and promoting gut function, this review provides a basis for the practical application of stilbenoids in animal health and husbandry.

## Simple Summary

The intestinal tract is vital for nutrient digestion, absorption, and immune defense in animals, and its health directly impacts growth and productivity. A healthy intestinal barrier, composed of physical, chemical, microbial, and immune components, facilitates nutrient absorption while preventing the infiltration of harmful substances like endotoxins and pathogens. Stilbenoids, such as resveratrol, pterostilbene, piceatannol, and oxyresveratrol, have shown great promise in supporting intestinal health. These compounds enhance gut morphology, strengthen intestinal barriers, and alleviate inflammation, contributing to improved animal growth and disease resistance. Additionally, stilbenoids modulate gut microbiota composition by promoting beneficial bacteria and suppressing harmful microbes, thus restoring microbial balance. This review highlights the multifaceted roles of stilbenoids in promoting gut health and provides the basis for their potential applications in improving animal health and animal husbandry practice.

## 1. Introduction

**Figure 1:**
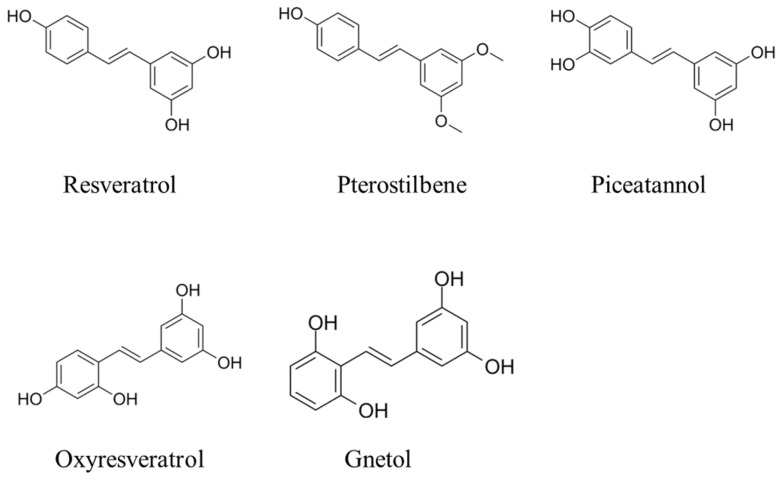
Chemical structures of selected stilbenoid phenolics showing common stilbene backbone.

Stilbenoids are an important class of phenolic compounds derived from plant families such as Vitaceae, Leguminaceae, Gnetaceae, and Dipterocarpaceae [1,2]. They share a common stilbene backbone but differ in substituent types and positions, resulting in diverse chemical structures and biological activities (Figure 1). As phytoalexins, stilbenoids such as resveratrol (RES), pterostilbene (PTE), oxyresveratrol (ORES), pinosylvin, viniferins, and piceatannol are known for their antimicrobial properties [3]. Beyond this, these compounds have also gained recognition for their potent antioxidant and anti-inflammatory activities, positioning them as promising candidates for improving animal health and productivity [4].

In livestock production, intestinal health is crucial for achieving optimal growth performance and disease resistance. The intestine not only facilitates nutrient absorption but also serves as a critical immune barrier, protecting against harmful pathogens and toxins. Disruption of this barrier can impair animal productivity, increase susceptibility to diseases, and elevate production costs. Thus, strategies to maintain intestinal integrity and improve gut function are of paramount importance.

This review aims to provide a comprehensive analysis of key stilbenoids, including RES, PTE, piceatannol, and ORES, with an emphasis on their biological effects and regulatory mechanisms in maintaining intestinal health. By examining their roles in mitigating inflammation-associated disorders and enhancing gut function, this review seeks to highlight the therapeutic potential of stilbenoids and identify directions for future research.

## 2. Chemistry of Stilbenoids

Stilbenoids are biosynthesized in plants’ response to biotic and abiotic stresses, including microbial infections, high temperatures, and oxidation. Functioning as phytoalexins, these compounds—such as RES, PTE, ORES, pinosylvin, viniferins, and piceatannol—play a pivotal role in plant defense mechanisms. By producing these polyphenolic substances, plants protect themselves against pathogens, including bacterial and fungal growth, and mitigate the damaging effects of UV radiation [5]. Structurally, stilbenoids are characterized by a C6–C2–C6 skeleton, consisting of an ethylene moiety flanked by two benzene rings, and they commonly exist in two isomeric forms. Representative examples of stilbenoids are illustrated in Figure 1. Although the presence of a double bond allows for both cis- and trans-forms, the trans-form is more stable and biologically relevant. Stilbenoids can occur as monomers or oligomers and may exist as free phenolic derivatives (aglycones) or as conjugated glucosides [3].

There is a significant interest in stilbenoids due to their numerous health-promoting properties, including antioxidant, anti-inflammation, antimicrobial, and anti-aging effects [4]. RES, in particular, has demonstrated antioxidant, immunomodulatory, anti-inflammatory, and antiangiogenic effects, offering a wide range of health benefits such as cancer chemoprevention and cardioprotection [6]. It is the most extensively studied stilbene, owing to its broad spectrum of biological activities [7,8]. Moreover, RES and its derivatives (Figure 1), including PTE, ORES, pinosylvin, viniferins, piceatannol, combretastatins, polydatin (piceid), mulberroside, and various oligostilbenes, are recognized for their diverse and potent biological activities and medicinal properties and have been the subject of extensive research [9,10,11].

## 3. Biological Effects of Stilbenoids

### 3.1. Anti-Inflammatory and Antioxidant

Anti-inflammatory and antioxidant activities form the cornerstone of the pharmacological benefits of stilbenoids. Among these, monomeric stilbenoids, such as RES, have been studied more extensively than their oligomeric counterparts due to their higher natural abundance and simpler structures, which facilitate easier identification and structural modifications for derivative development.

#### 3.1.1. RES

Resveratrol (RES, trans-3,5,4′-trihydroxystilbene) is a natural stilbenoid found in plants such as mulberries, peanuts, and grapes [6]. Known for its antioxidant, anti-inflammatory, and anti-aging properties, RES mitigates oxidative stress, regulates inflammatory responses, and protects against cellular damage, making it a key compound in promoting animal health [6].

RES exhibits significant antioxidative properties by enhancing the activities of antioxidant enzymes and reducing markers of oxidative damage. For instance, dietary supplementation with RES increased superoxide dismutase (SOD) and catalase (CAT) activities while reducing malondialdehyde (MDA) and hydrogen peroxide (H_2_O_2_) levels in the hearts of cold-exposed broilers, thus protecting heart tissues from oxidative stress [12]. Similarly, in Tibetan sheep, dietary RES and β-Hydroxy-β-methyl butyric acid supplementation significantly increased SOD and glutathione peroxidase (GSH-PX) activities in the liver [13]. These findings highlight RES’s ability to enhance systemic antioxidant defense, potentially benefiting various organisms under stress conditions.

RES also exerts potent anti-inflammatory effects through the regulation of multiple molecular pathways. Its anti-inflammatory activity is partly attributed to the inhibition of cyclooxygenase (COX)-1 and COX-2, reducing pro-inflammatory mediators such as nitric oxide (NO) and prostaglandin E2 (PGE2) [6,14]. In lipopolysaccharides (LPS)-stimulated RAW264.7 cells, cotreatment with RES-enriched rice seed extracts downregulated immune-associated genes and suppressed mitogen-activated protein kinase (MAPK) and nuclear factor kappa-B (NF-κB) signaling pathways [14]. Further studies revealed that RES inhibits NF-κB nuclear translocation and transcriptional activity by blocking the phosphorylation of its p65 subunit and inhibitor of kappa B alpha (IκBα) kinase [6]. Additionally, RES downregulates MAPKs such as ERK and p38 kinase, contributing to the inhibition of NF-κB signaling. This multifaceted mechanism underscores RES’s potential to mitigate inflammation in various contexts.

#### 3.1.2. PTE

Pterostilbene (PTE, 3,5-dimethoxy-4′-hydroxystilbene) is a natural antitoxin found in sources such as rosewood, grapes, blueberries, and other berries [15,16]. As a methylated derivative of RES, PTE replaces hydroxyl groups at the three and five positions with methyl groups, enhancing lipid solubility, membrane permeability, and metabolic stability, which collectively improve its bioavailability [15,17,18,19]. Compared to RES, PTE exhibits superior antioxidative and anti-inflammatory efficacy, underscoring its therapeutic potential [20].

Through the nuclear factor-erythroid 2 related factor 2 (Nrf2)/heme oxygenase-1 (HO-1) signaling pathway, PTE robustly activates antioxidant enzymes, thereby alleviating oxidative stress and apoptosis. For instance, PTE alleviates oxidative stress and ferroptosis in human granulosa cells by activating Nrf2/HO-1 [21]. As a strong activator of Nrf2, PTE increases the expression of antioxidant enzymes in a dose-dependent manner, protecting human keratinocytes against cytotoxicity and apoptosis induced by arsenic exposure [22]. In vitro studies show that PTE supplementation enhances the development rate of mouse blastocysts by modulating reactive oxygen species (ROS) and glutathione levels, reducing cellular turnover, and regulating ER stress-associated proteins [23]. Additionally, PTE significantly improves the antioxidant capacity of aging laying hens, evidenced by increased levels of glutathione, GSH-PX, SOD, CAT, and total antioxidant capacity (TAOC) in their ovaries, livers, and serum [24]. These findings highlight PTE’s potential in enhancing systemic antioxidant defense and combating oxidative stress.

Numerous studies demonstrate that PTE possesses robust anti-inflammatory properties. For example, PTE reduces the expression of pro-inflammatory cytokines such as interleukin (IL)-6 and tumor necrosis factor-α (TNF-α) in LPS-induced models, exhibiting anti-neuritis effects in rats [25]. In diabetic mice, PTE mitigates inflammatory responses by reducing the expression of IL-1β, TNF-α, and interferon-γ (IFN-γ), inhibiting inducible nitric oxide synthase (iNOS) activation, and decreasing NO formation in the pancreas [26]. Furthermore, PTE reduces the mRNA expression of IL-1α, IL-6, monocyte chemotactic protein-1 (MCP-1), and IL-1β, as well as NOD-like receptor thermal protein domain associated protein 3 (NLRP3) protein expression, both in vivo and in vitro, while inhibiting inflammatory cell migration and ROS production, suggesting its potential in preventing myocardial injuries and sepsis-induced complications [27]. Furthermore, PTE, alongside piceatannol and pinosylvin, suppresses carrageenan-induced paw inflammation in mice, reducing inflammatory edema and downregulating IL-6 and MCP-1 production. Remarkably, its effects were comparable to those of the commercial phosphatidylinositol 3-kinase (PI3K) inhibitor LY294002, suggesting that the anti-inflammatory effects of PTE may involve the inhibition of the PI3K/protein kinase B (Akt) pathway [28].

At the molecular level, PTE attenuates inflammation by modulating key signaling pathways. It inhibits the toll-like receptor (TLR)-NF-κB axis in high-fat diet-induced atherosclerotic mice, reducing adipose inflammation and downstream NF-κB p65 activity [29]. In co-culture systems of 3T3-L1 and RAW264.7 cells, PTE similarly downregulates the expression of inflammatory factors by inhibiting NF-κB activation [30]. Similarly, PTE suppresses LPS-induced neuroinflammation by inhibiting NF-κB and MAPK signaling pathways, including ERK1/2, c-Jun N-terminal kinase (JNK), and p38 [31]. Moreover, PTE regulates the TLR4/NF-κB pathway in both the colon and brain, reducing inflammatory factor release and alleviating intestinal and neuroinflammation [32]. Additionally, dietary supplementation with PTE activates the PI3K-Akt-mammalian target of the rapamycin (mTOR) signaling pathway, alleviating progressive oxidative stress and promoting placental nutrient transport in specific models [33]. Collectively, these findings highlight PTE’s multifaceted role in inflammatory regulation, spanning diverse biological systems and molecular pathways. Its ability to target upstream and downstream mediators of inflammation underscores its therapeutic potential in combating chronic inflammatory and oxidative stress-related diseases.

#### 3.1.3. Piceatannol

Piceatannol (trans-3,5,3′,4′-tetrahydroxystilbene) is a hydroxylated metabolite of RES, naturally found in foods such as berries, rhubarb, peanuts, passion fruit, white tea, and grape skin [34]. Its additional hydroxyl group at the 3′-position enhances antioxidative and anti-inflammatory properties by improving radical-scavenging ability, metabolic stability, and interactions with molecular targets [11,35,36]. These attributes underscore its therapeutic potential in addressing oxidative stress and inflammation.

Piceatannol demonstrates remarkably stronger antioxidant activity than RES, attributed to its additional hydroxyl group, which facilitates semiquinone radical formation [11,35,37]. It effectively reduces ROS generation and mitochondrial dysfunction in methylglyoxal-treated models [38]. Furthermore, piceatannol upregulates the Nrf2/HO-1 pathway, a key antioxidant mechanism. For instance, it induces HO-1 expression in endothelial cells through Nrf2 nuclear translocation [39,40]. The enhanced activation of Nrf2/HO-1 not only mitigates oxidative stress but also protects against homocysteine-induced apoptosis and endoplasmic reticulum (ER) stress [40]. A further study indicates that piceatannol not only promotes Nrf2/HO-1 expression but also inhibits the production of PGE2, NO, a disintegrin and metalloproteinase with thrombospondin motifs (ADAMTS5), and matrix metalloproteinases-13 (MMP13) [41]. Piceatannol’s antioxidant effects extend to various models. In PC-12 cells, it alleviates oxidative damage and mitochondrial dysfunction by modulating the sirtuin 3 (SIRT3) pathway [42]. In vivo, it reduces plasma lipid peroxidation and LDL cholesterol levels in metabolic models such as Zucker obese rats [43].

Piceatannol’s anti-inflammatory properties surpass those of RES, as evidenced by its inhibition of key pro-inflammatory mediators. It blocks NF-κB signaling by preventing inhibitor of kappa B kinase β (IKKβ) activation and suppressing phosphorylation of IκBα, thus reducing cytokine production (e.g., TNF-α, IL-6) and inhibiting COX-2 and iNOS expression [44,45,46,47]. In addition, piceatannol interferes with MAPK pathways, including p38 and JNK, further suppressing the activation of transcription factors like activator protein-1 (AP-1) [48]. This dual inhibition of NF-κB and MAPK pathways makes it a potent anti-inflammatory agent in various models, including mouse epidermis exposed to tumor promoters 12-O-tetradecanoylphorbol-13-acetate [48].

Moreover, its anti-inflammatory action extends to specific pathological conditions. For example, piceatannol protects against indomethacin-induced gastric ulcers through its antioxidative and anti-inflammatory effects [49]. Piceatannol’s antioxidative and anti-inflammatory activities intersect in its neuroprotective and cardioprotective roles. Through the SIRT3/FOXO3a pathway, it promotes mitophagy and alleviates oxidative damage in neuronal models, offering the potential for neurodegenerative disease treatment [50,51]. In the cardiovascular system, piceatannol regulates the PI3K-Akt-eNOS signaling pathway, mitigating oxidative stress and inflammation while enhancing endothelial function [52].

#### 3.1.4. ORES

ORES, trans-2,4,3′,5′-tetrahydroxystilbene, is an isomer of hydroxylated RES found in various plants, including pineapple honey (*Artocarpus lakoocha*), mulberry (*Morus alba L.*), Zingiberaceae, Verbascum (Liliaceae), *Trigonella foetidum* (Salviaceae), and Sarsaparilla (Sarsaparillaceae) [53,54,55]. As a natural stilbenoid, ORES shares similar biological activities with RES, but its additional hydroxyl group at the 2-position grants it enhanced antioxidant and anticancer effects [53]. Its strong antioxidant properties stem from multiple phenolic hydroxyl groups and superior tyrosinase inhibition [56,57]. ORES also exhibits anti-inflammatory, antioxidant, and neuroprotective effects [55,58].

ORES and its metabolites (e.g., glucuronides at the C-3 position) effectively reduce oxidative stress by decreasing the production of ROS and regulating intracellular calcium levels [59]. In rat cortical neurons, ORES protects against N-methyl-D-aspartate (NMDA)-induced toxicity by mitigating ROS generation and suppressing intracellular Ca^2+^ elevation [60]. ORES also demonstrates neuroprotective properties by enhancing mitochondrial function and reducing oxidative damage. For instance, in murine BV-2 microglial cells, ORES and its analogs suppress ROS and pro-inflammatory mediators via MAPK (ERK1/2, JNK, p38) and NF-κB signaling pathways [31].

ORES has shown efficacy in ameliorating inflammation in multiple in vivo models. In a mouse model of ethanol-induced gastric ulcers, ORES downregulated TNF-α, IL-6, NF-κB, and COX-2 expression while upregulating trefoil factor 2 (TFF-2) expression, demonstrating significant anti-inflammatory and immunomodulatory effects [61]. ORES exhibits greater inhibitory effects on NO, TNF-α, and COX-2 expression in macrophages and skin inflammation models than RES [31]. Its anti-inflammatory potency has been ranked as follows: PTE > ORES ≥ RES > acetyl-trans-resveratrol (ARES) ≥ trans-2,3,5,4′-tetrahydroxystilbene-2-O-glucopyranoside (TSG) [31]. Moreover, its ability to inhibit PGE2 production and NF-κB activity further underscores its robust anti-inflammatory profile [62]. In addition to its standalone efficacy, ORES exerts synergistic effects when combined with other natural compounds. For example, mulberry leaf extracts containing ORES and RES significantly suppressed inflammatory responses in LPS-stimulated macrophages, suggesting potential for combination therapies [63].

ORES modulates inflammation by targeting multiple pathways. In LPS-stimulated macrophages, ORES inhibits NF-κB activity and reduces iNOS and COX-2 expression, though its inhibition of NF-κB is weaker than RES due to its inability to form a semiquinone radical [47,59,63,64]. In addition to NF-κB, ORES targets the mitogen-activated extracellular signal-regulated kinase (MEK)/ERK pathway, specifically suppressing CXCR4-mediated activation in T cells, which reduces leukocyte migration and alleviates inflammation [63,65]. Moreover, ORES modulates the PI3K/Akt signaling pathway, ameliorating LPS-induced cognitive impairments and episodic memory deficits by downregulating inflammatory mediators such as TNF-α, IL-1β, iNOS, COX-2, and MMP9 [66]. Collectively, these mechanisms highlight ORES’s efficacy in mitigating inflammation and oxidative stress across various disease models. Its multifaceted regulatory effects make it a promising candidate for treating inflammation-related conditions and oxidative damage.

### 3.2. Antimicrobial Activity

Stilbenoids are secondary metabolites naturally found in plants like grapevines, where they act as phytoanticipins to prevent wood decay by inhibiting fungal pathogens [67]. In other tissues, their accumulation is triggered by microbial infection, including pathogens such as *Plasmopara viticola* and *Botrytis cinerea* [67,68].

RES and its derivatives exhibit significant inhibitory effects across a wide spectrum, including cocci, bacilli, and vibrio, with some antifungal activity as well. Specifically, it inhibits *Staphylococcus*, *Enterococcus*, and *Neisseria* within the genus Coccidioides, and shows antimicrobial activity against *Proteus mirabilis*, *Haemophilus ducreyi*, and *Helicobacter pylori* in the *Mycobacterium* genus [69,70]. Notably, RES protects host cells from inflammation and toxicity due to *Vibrio traumaticus* infections, with a minimum inhibitory concentration of 0.06 g/L for *Vibrio cholera* [71]. When combined with traditional antibiotics, it enhances the efficacy of aminoglycosides against *Staphylococcus aureus* but antagonizes the effects of fluoroquinolones on both *S. aureus* and *Escherichia coli* [72]. At sub-inhibitory concentrations, RES inhibits bacterial virulence factor expression, reduces toxin production and motility, and interferes with quorum sensing to prevent biofilm formation. Additionally, it demonstrates stronger inhibition against fungi such as *Botrytis cinerea* and *Trichophyton species*, while being less effective against *Fusarium tauroides* and *Candida albicans* [73]. These findings suggest that RES holds promise as a relatively safe antimicrobial agent.

Previous studies have shown that RES is not the most active stilbene in terms of antimicrobial activity [67,68]. While RES exhibits moderate antimicrobial properties, it serves as a precursor to more potent derivatives, such as PTE and viniferins. PTE, a derivative of RES, demonstrates superior antimicrobial activity due to its lower hydrophilicity and higher bioavailability. It efficiently penetrates biological membranes and disrupts biofilms, making it a promising candidate for treating biofilm-associated infections caused by *Staphylococcus* spp., *Enterococcus faecalis*, and *Bacillus cereus* [74]. PTE shows stronger antifungal activity against crop pathogens, including *Botrytis cinerea*, *Leptosphaeria maculans*, *Ginnia viticola Fusarium*, *Fusarium oxysporum*, *Sclerotinia sclerotiorum*, *Staphylococcus aureus*, and *Saccharomyces cerevisiae* [75,76]. Its enhanced efficacy is attributed to the presence of methoxy groups, which increase hydrophobicity and membrane permeability, enabling better interaction with lipophilic fungal membranes [77].

ORES demonstrated weak antibacterial activity in vitro against six standard strains and two clinical isolates of *Staphylococcus aureus* [78]. However, it showed notable inhibitory effects against methicillin-resistant *Staphylococcus aureus* and exhibited synergistic actions with ciprofloxacin and gentamicin by increasing bacterial cell membrane permeability and inhibiting ATPase activity [79]. A preliminary study indicated ORES’s antibacterial activity against *Staphylococcus aureus*, *Bacillus subtilis*, *Micrococcus flavus*, *Streptococcus faecalis*, *Salmonella abony*, and *Pseudomonas aeruginosa* [80]. It also inhibited *uropathogenic Escherichia coli* by suppressing biofilm formation, swarming motility, fimbriae production, and hemagglutination ability [81].

Furthermore, ORES affected quorum sensing in *Chromobacterium violaceum* CV026 by inhibiting bacterial signaling, as evidenced by reduced violacein production [82]. In *Pseudomonas aeruginosa* PAO1, ORES decreased pyocyanin production and swarming motility without effects on bacterial growth [82]. ORES also exhibited antifungal effects against several dermatophytes, including *Trichophyton rubrum*, *Trichophyton mentagrophytes*, *Trichophyton tonsurans*, *Microsporum canis*, *Microsporum gypseum*, and *Epidermophyton floccosum*, and showed synergistic effects with the antifungal drug miconazole nitrate against *T. rubrum* [65,83]. Studies on the heartwood of osage orange (Maclura pomifera) revealed that ORES is produced in response to wood-decaying fungi such as *Trametes versicolor* and *Gloeophyllum trabeum*, inhibiting the wood-degrading enzyme glutathione transferase Omega found in Trametes versicolor [84].

## 4. Multifaceted Roles of Stilbenoids in Gut Health and Function

Environmental stress and other factors during early growth can compromise the intestinal barrier in animals, resulting in underdeveloped immune function and imbalanced intestinal flora [85]. Such disruptions not only increase susceptibility to various diseases but also negatively impact production performance [8]. The intestinal barrier, consisting of physical, chemical, microbial, and immune components, serves as a critical interface between the internal environment and external stimuli. It plays an essential role in facilitating nutrient absorption while preventing harmful substances, such as endotoxins and pathogenic microorganisms, from breaching the epithelium and infiltrating internal tissues [86].

Given the significance of intestinal health, growing evidence highlights that stilbenoids, including RES, PTE, and ORES, offer significant benefits in protecting intestinal health and alleviating inflammatory bowel disease (IBD) [87,88]. These compounds enhance gut morphology and mucosal function by improving tight junction integrity and reducing inflammation [89]. They also modulate gut microbiota composition, promoting beneficial bacteria and suppressing harmful taxa to restore microbial balance [90]. Furthermore, stilbenoids influence key metabolic pathways, such as short-chain fatty acids (SCFAs) production, while maintaining mitochondrial function to reduce oxidative stress and support cellular energy needs [91,92]. These mechanisms collectively highlight their therapeutic potential for preserving intestinal barrier integrity and promoting gut homeostasis. The following sections delve into their mechanisms of action and therapeutic potential, as demonstrated in animal models and cell-based studies.

### 4.1. Enhancement of Gut Morphology and Mucosal Function and Related Intestinal Inflammation

A complete intestinal epithelial barrier is essential for maintaining normal physiological functions. When this barrier is compromised, harmful substances like bacteria and toxins from the intestinal lumen can penetrate epithelial tissue, triggering inflammatory reactions and potentially leading to IBD. Stilbenoids, particularly RES and its derivatives, have been extensively studied for their therapeutic effects on intestinal inflammatory reactions. In IBD mouse models, RES demonstrated anti-inflammatory properties by increasing IL-10 levels and reducing pro-inflammatory cytokines such as TNF-α, IL-6, IL-1β, and IL-8 [87]. Similarly, treatment with RES and PTE at 5% concentrations downregulated duodenal brush border proteins, including aminopeptidase, IL-1β, and TNF-α, with PTE also reducing IL-6 levels [88]. Moreover, RES alleviates colitis symptoms by enhancing the expression of tight junction proteins (TJPs), including Occludin and Claudin 1, while increasing anti-inflammatory cytokine IL-10 and reducing pro-inflammatory cytokines IL-1β, IL-6, and TNF-α [93]. These findings collectively highlight RES’s capacity to reduce intestinal mucosal inflammation, repair tissue damage, and restore tight junction integrity.

**Figure 2:**
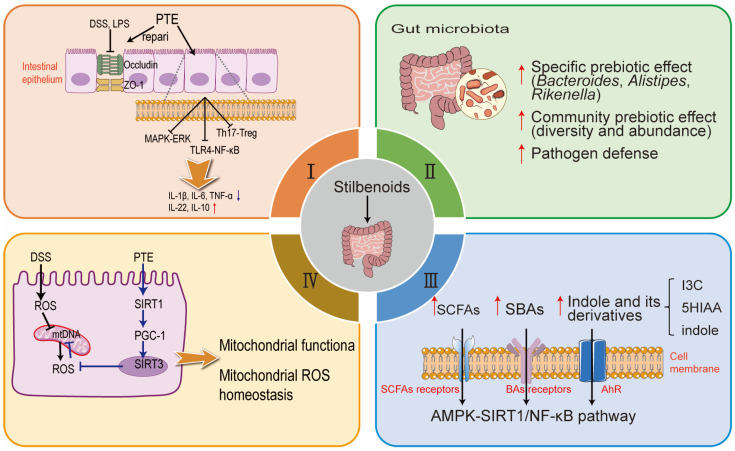
Multifaceted roles of stilbenoids in gut health and function (RES and PTE as an example). I. PTE alleviates intestinal barrier injury and intestinal inflammation induced by DSS or LPS. II. RES improves intestinal microorganisms. III. RES enhances intestinal microbial metabolites and acts on AMPK-SIRT1/NF-κB pathway. IV. PTE inhibits DSS-induced ROS production via the SIRT1-PGC-1- SIRT3 pathway, thereby maintaining mitochondrial ROS homeostasis and sustaining mitochondrial function. Key abbreviations: DSS, dextran sulfate sodium; LPS, lipopolysaccharides; ZO-1, zonula occludens-1; SCFAs, short-chain fatty acids; SBAs, secondary bile acids; I3C, indole-3-carbinol; 5HIAA, 5-hydroxyindole-3-acetic acid; mtDNA, mitochondrial DNA; SIRT1, sirtuin 1.

Similarly, PTE exhibits significant potential in preventing colitis by modulating inflammation, fibrosis, and gut barrier function (Figure 2). Dietary supplementation with PTE at 0.005–0.025% effectively reversed colitis symptoms, reducing the colon weight-to-length ratio, aberrant crypt foci, and colonic wall thickening [94]. PTE also decreased markers of fibrosis, including transforming growth factor-β1 (TGF-β1), COX-2, and p-Smad2, which are central to the TGF-β1/Smad signaling pathway [94]. In models of diabetes, LPS, or dextran sulfate sodium (DSS)-induced enteritis models, PTE mitigated oxidative stress, reduced inflammatory markers, and preserved essential gut components such as E-cadherin and Muc2-coated cells [26,95,96]. Its beneficial effects on gut health were further attributed to reduced ER stress and ferroptosis regulation, as well as suppression of macrophage S100A8-TLR-4-NF-κB signaling cascades [97,98]. Reduced phosphorylation of p38 MAPK and ERK1/2 may also contribute to PTE’s beneficial effects on gut barrier integrity [89]. In DSS-induced colitis models, oral administration of PTE not only alleviated colitis symptoms but also significantly reduced TNF-α expression in mice. The protective effects of PTE in DSS-induced colitis appear to be mediated by the inhibition of T helper cell (Th) 1/Th17 pathways and the promotion of regulatory T cells (Treg) [99]. Collectively, these findings underline PTE’s multifaceted role in gut barrier protection and inflammation reduction.

Finally, ORES has emerged as another promising agent for improving gut permeability and inflammation. Studies in Caco-2 and goblet cells revealed that ORES increases TJPs and mucin production, likely through activation of protein kinase C (PKC) and MAPK pathways, as well as upregulation of trefoil factor 3 [100,101]. In DSS-induced colitis models, ORES reduced disease activity index scores, preserved goblet cell numbers, and modulated cytokine expression to protect colonic structures [102]. Its anti-inflammatory properties were also demonstrated in ethanol-induced ulcer models, where ORES decreased pro-inflammatory markers like IL-6, TNF-α, NF-κB, and COX-2 levels, without affecting COX-1 levels [61]. Although ORES exhibits antioxidant and mucosal protective effects, recent studies suggest it may act as a pro-oxidant in the presence of Cu^2+^ ions, highlighting the need for further investigation into its mechanisms [103].

### 4.2. Modulation of Gut Microbiota Composition

The gut microbiota is integral to maintaining host immune function, organ development, and overall physiology. Central to this system is the intestinal biological barrier, predominantly comprised of beneficial anaerobic bacteria such as *Lactobacillus* and *Bifidobacterium*. These microbes contribute to gut health by secreting organic acids—such as lactic, acetic, propionic, and butyric acids—that lower intestinal pH and inhibit the growth of pathogenic bacteria. Moreover, the interplay between the intestinal epithelial barrier, dietary nutrients, and microbiota reflects the complex regulatory mechanisms required for gut homeostasis [104].

RES and its derivatives, known for their antioxidant, anti-inflammatory, and antibacterial properties, face bioavailability challenges due to poor solubility and rapid metabolism [105]. Following ingestion, approximately 77–80% of resveratrol is absorbed in the intestine, with blood concentrations peaking around 60 min [105]. These limitations have redirected research toward their interactions with gut microbiota, as a significant portion of stilbenoids undergo metabolism in the gastrointestinal tract [106]. Emerging evidence indicates that stilbenoids influence gut microbial composition, notably reducing the *Firmicutes*-to-*Bacteroidetes* ratio and decreasing harmful bacteria such as *Clostridium* strains while promoting beneficial species like *Faecalibacterium prausnitzii* [107]. Notably, dietary RES has demonstrated its ability to restore microbial diversity in colitis models (Figure 2). For instance, it significantly decreased pro-inflammatory genera such as *Akkermansia*, *Dorea*, *Sutterella*, and *Bilophila* while increasing beneficial *Bifidobacterium* in DSS-treated mice [90]. In db/db mice, RES reversed dysbiosis characterized by reductions in beneficial genera such as *Bacteroides*, *Alistipes*, and *Rikenella*. Furthermore, fecal microbiota transplantation from RES-treated mice significantly alleviated gut inflammation and improved intestinal health in recipient mice [91]. These findings highlight the pivotal role of RES in maintaining gut microbial homeostasis and mitigating intestinal inflammation.

RES has also demonstrated notable benefits in improving intestinal health under oxidative stress conditions by modulating the gut microbiome. In diquat-challenged piglets, RES supplementation reduced the abundance of taxa such as *Firmicutes*, *Actinobacteria*, *Ruminococcaceae UCG-005*, and *Eubacterium coprostanoligenes*, while promoting beneficial bacteria like *Clostridium sensu stricto 1* and *Lachnospiraceae unclassified* [108]. Furthermore, RES significantly improved gut microbial diversity and reversed alterations in key phyla, including *Bacteroidetes*, *Proteobacteria*, and *Firmicutes*. It increased the presence of beneficial genera and suppressed potential pathogens, such as *Lachnoclostridium*, *Acinobacter*, and *Serratia*, thereby restoring intestinal microbial balance [93]. These results emphasize the potential of RES in mitigating oxidative stress-induced dysbiosis and promoting gut health.

Both PTE and RES play pivotal roles in remodeling the gut microbiome, enhancing microbial diversity, and promoting the growth of beneficial bacteria, such as *Blautia* and *Lachnospiraceae UCG-001*, while suppressing harmful species like *Eubacterium ventriosum* and *Acetitomaculum* [109]. These effects contribute to alleviating intestinal morphological abnormalities, improving intestinal permeability, and correcting dysregulation associated with intrauterine growth retardation in piglets. Notably, PTE has demonstrated greater efficacy than RES in combating oxidative stress under these conditions [110]. Furthermore, PTE exhibits unique antimicrobial properties, such as reducing the cell viability of *Bacillus cereus*, while concurrently benefiting the gut microbiota by increasing the abundance of beneficial *Bacteroidetes* and reducing pathogenic taxa, including *Firmicutes*, *Helicobacter*, *Desulfovibrio*, *Lachnospiraceae*, and *Mucispirillium* [111,112]. It also enhances intestinal flora homeostasis, boosts SCFA levels, and inhibits the loss of TJPs, mitigating intestinal inflammation and barrier damage [32]. Taken together, these findings highlight the complementary roles of PTE and RES in modulating gut microbiota composition, enhancing intestinal integrity, and alleviating inflammation. Their ability to remodel microbial communities, restore gut health, and address intestinal disorders underscores their therapeutic potential for maintaining a balanced and resilient gut microbiome.

### 4.3. Regulation of Metabolic Pathways and Gut Metabolome

Variations in microbiota composition significantly influence the production of microbial metabolites, which in turn impact host health positively or negatively [113,114]. For instance, metabolites such as SCFAs, bile acids (BAs), and tryptophan derivatives play crucial roles in maintaining intestinal health by enhancing mucosal integrity and modulating immune responses. Reduced levels of SCFAs, indole derivatives, and secondary bile acids (SBAs) have been observed in patients with IBD, correlating with increased disease severity. Supplementation of these metabolites alleviates experimental colitis in mouse models [115].

Given the importance of metabolites such as SCFAs for intestinal health, stilbenes represented by RES may be an effective intervention strategy by promoting the production of probiotics and related metabolites (Figure 2). RES supplementation influences gut microbiota composition by promoting beneficial bacteria such as *Parabacteroides* and *Alistipes*, which are linked to reduced inflammation and enhanced SCFA production [91,92]. RES supplementation significantly increased diquat-induced some metabolites including indole-3-carbinol, 5-hydroxyindole-3-acetic acid, indole, and alpha- and beta-dihydroresveratrol, and uridine [108]. Additionally, RES-derived microbial metabolites, such as 3-(4-hydroxyphenyl)-propionic acid (4HPP), exhibit anti-inflammatory effects and improve intestinal barrier function through mechanisms involving the AMP-activated kinase (AMPK)-SIRT1/NF-κB pathway [116]. These findings suggest that RES can exert its protective effects in part by modulating microbial metabolites critical for gut health.

Recent studies have highlighted significant changes in key metabolic pathways in response to therapeutic interventions. For example, based on spatial metabolomics techniques, PTE has demonstrated therapeutic potential in cerebral ischemia–reperfusion injury by modulating disrupted small-molecule metabolic pathways, including energy supply, neurotransmitter synthesis, polyamine levels, and phospholipid metabolism [117]. Restoration of creatine and creatinine levels further underscores its role in enhancing energy storage and supply. Additionally, creatine, creatinine, sarcosine, and 4-acetamidobutanoate are pivotal in the arginine and proline metabolic pathways. RES targets this pathway, contributing to reduced colitis severity by modulating key metabolic functions [93].

### 4.4. Maintenance of Mitochondrial Health and Oxidative Balance

Maintaining mitochondrial health is critical for preserving the epithelial barrier’s integrity, which includes tight junctions, mucus production, antimicrobial peptides, and immune tolerance [118,119,120]. This is particularly important because enterocytes, the intestinal epithelial cells, are rich in mitochondria to meet the intestine’s high energy demands. As a vital organ for digestion and metabolism, the intestine requires substantial energy to support its self-renewal and functionality. Moreover, studies in conplastic murine lines carrying mitochondrial DNA (mtDNA) polymorphisms have shown that enhanced oxidative phosphorylation efficiency, leading to higher intestinal ATP production, can protect against DSS-induced colitis [120]. Similarly, mitophagy, a process essential for maintaining mitochondrial quality, helps control inflammation and safeguard tight junction integrity [121,122]. Conversely, impaired mitophagy disrupts mitochondrial function, reduces energy production, and compromises the epithelial barrier.

In IBD, electron transport chain deficits/damage result in elevated mitochondrial ROS production, which damages mtDNA and other critical components, further amplifying ROS accumulation [120,123,124,125,126]. This oxidative stress exacerbates mitochondrial dysfunction, contributing to intestinal epithelial barrier damage. Mouse models demonstrate that pharmacological induction of mitochondrial ROS can trigger colitis, whereas ROS inhibition alleviates disease severity, highlighting the pathological role of excessive ROS [127]. To counteract oxidative stress, mitophagy plays a protective role by removing damaged mitochondria, thereby limiting ROS accumulation and maintaining mitochondrial quality. This process also inhibits apoptosis by reducing mitochondrial outer membrane permeabilization and preventing the release of pro-apoptotic proteins [128]. Supporting this, Nix−/− mice, which lack the mitophagy receptor Nix/Bnip3l, are more susceptible to DSS-induced colitis and show greater mitochondrial damage in colonic epithelial cells [129]. Importantly, treatment with Mito-Tempo, a mitochondrial-targeted ROS scavenger, mitigates DSS-induced colitis and upregulates NIX expression, further emphasizing the therapeutic potential of targeting mitochondrial ROS.

Therapeutic interventions such as RES and PTE have shown promising potential in protecting intestinal epithelial cells from oxidative stress and mitochondrial dysfunction. For instance, RES (0.1 mg/mL) protects intestinal epithelial Caco-2 cells from indomethacin-induced mitochondrial dysfunction [130] and mitigates IS-induced mitophagy impairment and intestinal epithelial damage by restoring the IRF-DRP1 axis [131]. Meanwhile, RES enhances mitophagy to remove damaged mitochondria caused by the diquat challenge [132]. Similarly, PTE enhances mitophagy by upregulating PTEN-induced putative kinase 1 (PINK1), Parkin, and LC3B expression [133]. Both compounds effectively enhance mitochondrial antioxidant capacity, alleviate oxidative stress-induced injury, and reduce apoptosis by modulating key pathways, including SIRT1 signaling. Notably, PTE appears to be more efficacious than RES in preserving intestinal integrity and mitochondrial function, as demonstrated in various in vitro and in vivo models [134]. Mechanistically, RES and PTE mitigate oxidative stress by enhancing the expression of SIRT3 and promoting the deacetylation of key mitochondrial antioxidant enzymes, such as SOD2 and peroxiredoxin 3. In H_2_O_2_-exposed IPEC-J2 cells, RES and PTE reduced proliferator-activated receptor gamma coactivator 1-alpha (PGC-1α) acetylation, leading to increased mitochondrial transcription factor A protein levels and mtDNA copy numbers. This process reduces mitochondrial ROS levels, stabilizes mitochondrial membrane potential, and prevents the release of cytochrome C and subsequent caspase-3 activation. In H_2_O_2_-exposed IPEC-J2 cells, these effects were abolished upon SIRT1 depletion, underscoring the critical role of SIRT1 in mediating the protective effects of RES and PTE [20] (Figure 2). Given their multifaceted protective mechanisms, RES and PTE hold great potential for therapeutic applications in oxidative stress-related intestinal disorders. Future studies should explore their long-term safety and efficacy in husbandry, as well as investigate the precise molecular interactions within pathways such as protein kinase A (PKA) PKA/liver kinase B1 (LKB1)/AMPK and SIRT1.

## 5. The Potential of Stilbenoids in Enhancing Livestock Gut Health

Emerging evidence highlights stilbenoids, particularly RES and PTE, as a promising solution to support gut health through multiple mechanisms (Table 1). These compounds have demonstrated their ability to mitigate stress-induced damage, enhance gut morphology, and improve microbial homeostasis in livestock. For instance, RES supplementation in piglets under oxidative stress has been shown to modulate gut microbiota composition and metabolite profiles, contributing to improved intestinal health [108]. Similarly, in deoxynivalenol-challenged piglets, RES reduces D-lactate levels and pro-inflammatory markers (TNF-α, IL-1β) while increasing zonula occludens-1 (ZO-1) expression, enhancing gut barrier integrity [135].

PTE has demonstrated significant benefits in broiler chickens experiencing immunological stress by alleviating intestinal inflammation, preserving mucosal integrity, and promoting growth performance [96]. In broilers exposed to diquat-induced stress, PTE further strengthens gut barrier function by enhancing antioxidant capacity and regulating ferroptosis [97]. Stilbenoids have also been linked to improved micronutrient absorption, as demonstrated by their effects on duodenal morphometry following intra-amniotic administration [88].

Most of these studies were conducted in livestock, including broiler chickens [96,97], weaning piglets [136,137], and Cornish cross-fertile broilers [88], with some research also involving stressed animal models, such as heat-stressed ducks [138]. Collectively, the findings underscore the efficacy of stilbenoids in improving intestinal health, strengthening the gut barrier, and mitigating oxidative stress and inflammation in livestock. These results not only highlight the potential of stilbenoids in addressing gut-related challenges but also pave the way for future research to fully harness their therapeutic benefits in animal health and production.

**Table 1:** Effects of stilbenoids in various animal models.

Stilbenoids	Concentration	Model System	Observations	Ref.
RES	90 mg/kg	Piglets under oxidative stress	↑The genera *Clostridium sensu stricto 1* and *Lachnospiraceae unclassified*, ↑indole-3-carbinol, ↑5-hydroxyindole-3-acetic acid, and ↑uridine	[108]
RES	300 mg/kg	Deoxynivalenol-challenged piglets	↓D-lactate levels, ↓pro-inflammatory markers (TNF-α, IL-1β), and↑ZO-1 expression	[135]
RES	500 mg/kg	Cold-exposed broilers	↑The activities of SOD and CAT and mRNA expression of anti-inflammatory genes, ↓concentrations of MDA and H_2_O_2_ and mRNA expression of ER stress, pyroptosis and proinflammatory genes	[12]
RES	100 mg/kg	Diquat challenged piglets	↑occludin, claudin-1, and ZO-1 proteins, improved redox status, ↓mitochondrial damage, and induced mitophagy	[132]
RES	90 mg/kg	Diquat-challenged piglet	Protected intestinal integrity, ↓oxidative stress, and↑Akt/Nrf2 signaling pathway	[139]
RES	400 mg/kg	Acute heat stressed ducks	↑Villus height to crypt depth ratio, ↑goblet cell number, ↓histopathological damage in jejunum, and↑SIRT1 signaling pathway	[138]
RES	400 mg/kg	LPS-induced broilers	↑Average daily gain, ↓spleen index, ↓IgM, ↓secretory immunoglobulin A levels; ↓D-lactic acid, ↑occludin mRNA expression, ↓TLR4, ↓NF-κB, and ↓TNF-α levels	[140]
RES	400 mg/kg	Heat stressed broilers	↑Body weight, ↑average daily gain, ↑relative jejunum weight and length, ↑villus height, ↑GPX and glutathione S-transferase activities, ↑Nrf2 and SOD1 mRNA levels, and ↓Keap1 mRNA expression	[141]
RES	150 and 300 mg/kg	Weaning piglets	↑Serum IgG, ↑GPX activity, ↓MDA content, ↑villus height to crypt depth ratio, ↑jejunum villus height, ↓crypt depth, and ↑IL-10 and ZO-1 mRNA levels	[136]
RES	300 mg/kg	Weaning piglets	↑The proportion of butyrate-producing bacteria, include *Flavonifractor*, *Odoribacter*, and *Oscillibacter*	[137]
PTE	400 mg/kg	Broiler chickens with immunological stress	↑Body weight, ↑villus height to crypt depth ratio, ↑ZO-1 and occluding mRNA levels, and ↓the nuclear translocation of NF-κB p65	[96]
PTE	400 mg/kg	Broilers under diquat-induced stress	↓Intestinal permeability, ↓jejunal apoptosis rate, ↑jejunal villus height, ↑villus height to crypt depth ratio, ↓ROS, ↑SOD2, ↑occludin, ↑ZO-1, and ↑Nrf2 pathway activation	[97]

Footnote: “↑” represents “increase”, “↓” represents “decrease”.

## 6. Summary and Perspectives

Stilbenoids, such as RES and PTE, exhibit significant antioxidative and anti-inflammatory effects through mechanisms involving the Nrf2/HO-1, PI3K/Akt, TLR-NF-κB, and MAPK signaling pathways. These mechanisms, coupled with their roles in enhancing gut morphology, modulating gut microbiota composition, supporting mucosal immune responses, and regulating metabolic pathways, underscore their therapeutic potential in maintaining gut health and addressing intestinal disorders. Animal studies have demonstrated that stilbenoids can improve growth performance, alleviate stress-induced intestinal damage, and enhance immune function, suggesting promising applications in animal husbandry.

Despite the extensive research on RES, the therapeutic potential of less common stilbenoids, such as PTE, piceatannol, and gnetol, remains underexplored. Future studies should prioritize their specific biological effects, dose–response relationships, and safety profiles in different animal models. Research on synergistic effects and the comparative efficacy of different stilbenoids is currently limited, but addressing these gaps could significantly advance the field. Additionally, challenges such as limited bioavailability and the lack of long-term toxicity data warrant further investigation. Specific dosages, as provided in Table 1, offer a starting point for practical applications, but further research into dosage optimization, long-term safety, and the combinatorial effects of stilbenoids with other gut health-promoting compounds is essential. These efforts will pave the way for the practical application of stilbenoids in promoting animal health and productivity.

## Abbreviations

RES: resveratrol; PTE, pterostilbene; ORES, oxyresveratrol; SOD, superoxide dismutase; CAT, catalase; MDA, malondialdehyde; H_2_O_2_, hydrogen peroxide; GSH-PX, glutathione peroxidase; COX, cyclooxygenase; NO, nitric oxide; PGE2, prostaglandin E2; LPS, lipopolysaccharides; MAPK, mitogen-activated protein kinase; NF-κB, nuclear factor kappa-B; IκBα, inhibitor of kappa B alpha; ERK, extracellular regulated protein kinases; Nrf2, nuclear factor-erythroid 2 related factor 2; HO-1, heme oxygenase-1; ROS, reactive oxygen species; TAOC, total antioxidant capacity; IL, interleukin; IFN-γ, interferon-γ; iNOS, inducible nitric oxide synthase; MCP-1, monocyte chemotactic protein-1; NLRP3, NOD-like receptor thermal protein domain associated protein 3; PI3K, phosphatidylinositol 3-kinase; Akt, protein kinase B; TLR, toll-like receptor; JNK, c-Jun N-terminal kinase; mTOR, mammalian target of rapamycin; ER, endoplasmic reticulum; MMP13, matrix metalloproteinases-13; ADAMTS5, a disintegrin and metalloproteinase with thrombospondin motifs; SIRT3, sirtuin 3; IKKβ, inhibitor of kappa B kinase β; AP-1, activator protein-1; NMDA, N-methyl-D-aspartate; TFF-2, trefoil factor 2; ARES, acetyl-trans-resveratrol; TSG, trans-2,3,5,4′-tetrahydroxystilbene-2-O-glucopyranoside; MEK, mitogen-activated extracellular signal-regulated kinase; IBD, inflammatory bowel disease; SCFAs, short-chain fatty acids; TJPs, tight junction proteins; TGF-β1, transforming growth factor-β1; DSS, dextran sulfate sodium; Th, T helper cell; Treg, regulatory T cells; PKC, protein kinase C; BAs, bile acids; SBAs, secondary bile acids; 4HPP, 3-(4-hydroxyphenyl)-propionic acid; AMPK, AMP-activated kinase; mtDNA, mitochondrial DNA; PINK1, PTEN induced putative kinase 1; PGC-1α, proliferator-activated receptor gamma coactivator 1-alpha; ZO-1, zonula occludens-1; IgM, ImmunoglobulinM; PKA, protein kinase A; LKB1, liver kinase B1.
